# Firearm-related experiences and perceptions among United States male veterans: A qualitative interview study

**DOI:** 10.1371/journal.pone.0230135

**Published:** 2020-03-10

**Authors:** Joseph A. Simonetti, Brooke Dorsey Holliman, Ryan Holiday, Lisa A. Brenner, Lindsey L. Monteith

**Affiliations:** 1 Rocky Mountain Mental Illness Research, Education and Clinical Center for Suicide Prevention, Rocky Mountain Regional VA Medical Center, Veterans Health Administration, Aurora, Colorado, United States of America; 2 Seattle-Denver Center of Innovation for Veteran-Centered and Value-Driven Care, Veterans Health Administration, Aurora, Colorado, United States of America; 3 Hospital Medicine Group, Rocky Mountain Regional VA Medical Center, Aurora, Colorado, United States of America; 4 Department of Community and Behavioral Health, University of Colorado Anschutz School of Public Health, Aurora, Colorado, United States of America; 5 Department of Psychiatry, University of Colorado Anschutz Medical Campus, Aurora, Colorado, United States of America; 6 Department of Physical Medicine and Rehabilitation, University of Colorado Anschutz School of Medicine, Aurora, Colorado, United States of America; 7 Department of Neurology, University of Colorado, Anschutz Medical Campus, Aurora, Colorado, United States of America; University of California San Diego School of Medicine, UNITED STATES

## Abstract

**Background/Objective:**

Male veterans ages 55–74 comprise a disproportionate number of suicide deaths among United States veterans, for whom a majority of suicides are firearm-related. Little is known about the firearm-related experiences and beliefs of veterans, which could be informative for firearm-related lethal means safety interventions. The aim of this qualitative study was to identify themes relevant to developing such interventions among older male veterans.

**Methods:**

We conducted semi-structured qualitative interviews with seventeen United States male veterans, ages 50–70, who were eligible to receive Veterans Health Administration services, and were current or former firearm owners or users. Transcripts were analyzed via thematic analysis using an inductive approach.

**Results:**

Six themes were identified: 1) Firearm experiences were usually facilitated by male family members and occurred at an early age; 2) Safety lessons during early firearm encounters focused on preventing unintentional injuries through safe firearm handling and using “common sense;” 3) Firearms serve an important social function across veterans’ lifespans (e.g., hunting with friends); 4) Veterans perceive firearms as useful for protection; 5) Veterans believe that not everyone should have access to firearms, and some described scenarios in which they acted to limit others’ access during unsafe situations; and 6) Veterans have preferences for who is involved in firearm safety discussions.

**Conclusions:**

We identified themes relevant to developing firearm-specific lethal means safety interventions among older male veterans. Findings suggest potential obstacles (e.g., sociocultural value of firearms) to affecting changes in firearm behaviors, and factors that could potentially facilitate interventions (e.g., family involvement). Consideration of these findings may be important for developing personalized, effective interventions for this population.

## Introduction

Suicide risk is higher among United States (U.S.) veterans relative to non-veteran adults [1, 2]. A disproportionate number of veteran suicides occur among males ages 55–74 years, and the suicide rate increased among this cohort from 2005–2017 [2]. In 2017, 71% of veteran male suicides were firearm-related in comparison to 54% of non-veteran, adult male suicides [1, 2]. Given that firearm access is independently associated with suicide risk, this difference is likely in part attributable to the higher prevalence of firearm ownership among male veterans in comparison with non-veteran, adult males (47% vs. 30%) [3, 4].

Access to a household firearm or the unsafe storage of firearms are associated with a higher risk of suicide [4–6]. As such, efforts to promote reducing access to firearms among those with elevated suicide risk, known as lethal means safety, is considered an important element of suicide prevention programs [7, 8]. Approximately one in three veteran firearm owners store at least one household firearm loaded and unlocked [9]. Among veterans receiving Veterans Health Administration (VHA) services, 45% of those with mental health risk factors for suicide (e.g., depression) store at least one firearm loaded and unlocked [10].

Several healthcare systems, including VHA, have expanded efforts to promote firearm-related lethal means safety [11, 12]. However, of the interventions known to be effective in promoting safe firearm storage, none have been implemented to prevent firearm-related suicide among veterans or non-veteran adults [13]. As such, little is known about challenges to promoting firearm-related lethal means safety in clinical settings among these populations. Furthermore, knowledge is lacking regarding the experiences and beliefs of firearm users and how they may influence safety behaviors, risk perceptions, and firearm-related lethal means safety interventions.

In prior studies among adults, including firearm owners and veterans, participants have generally agreed that clinic-based firearm-related discussions are appropriate under some circumstances [14–17]. For example, in a survey study conducted among a nationally-representative sample of U.S. adults in 2015, 66% of adults, 57% of veterans, and 54% of firearm owners said that it is “*at least sometimes appropriate*” for providers to talk to patients about firearms [15]. In studies conducted among VHA mental health patients and stakeholders (e.g., clinicians, family), most have agreed that is acceptable to implement firearm-related lethal means safety interventions in clinical settings [16, 17], particularly for patients with mental health diagnoses [17]. However, preferences for whom should initiate or be involved in such discussions remains unclear. In a focus group study conducted among veterans, many suggested that involving family and/or friends in such conversations is important [17]. In contrast, in a nationally-representative study of firearm owners conducted in 2016, only 19% of respondents agreed that physicians would be “*good*” or “*excellent*” messengers to teach patients about safe firearm storage [18]. Thus, while many veterans and firearm owners agree that delivering firearm safety interventions in clinical settings is appropriate, it is unclear under which circumstances patients might find such discussions or certain messengers acceptable.

Risk perceptions strongly influence risk behaviors [19], yet the specific circumstances in which individuals consider firearm access to be a risk factor for suicide are unclear. For example, only 6% of U.S. veterans and non-veteran adults agree that firearm access increases suicide risk for household members [9, 20]. Yet, when asked what actions they would take if a household member were to become suicidal, 82% of veterans reported they would limit that individual’s access to firearms [10]. The reasons individuals choose to own or use firearms may be also be important barriers to behavior change. Two-thirds of veterans and non-veteran adults who own firearms report having them for personal or household protection [3, 21], and those who have firearms for protection are more likely to store their firearms unlocked and loaded with ammunition [10]. Thus, decisions about reducing one’s suicide risk by securing or removing household firearms may be influenced by a perceived trade-off in increasing vulnerability to victimization by others. However, the specific reasons that firearm owners feel the need for protection have not been well-characterized, including among veterans.

To inform development and implementation of effective firearm-related suicide prevention interventions among older male veterans, it is essential to improve our understanding of their firearm-related experiences and perspectives. In this qualitative study, we aimed to identify themes relevant to developing and implementing firearm-specific lethal means safety interventions among older U.S. male veterans.

## Methods

### Participants and recruitment

We included U.S. male veterans who were 50–70 years of age, eligible to receive VHA services, and were current or former firearm owners or users. We included former firearm owners and users as their perspectives might be informative for understanding why veterans decide to forgo firearm ownership or use, and that firearm-related suicide risk is not unique to those who own firearms. We excluded those unable to provide informed consent or with severe psychiatric symptoms precluding study participation. We recruited participants by posting fliers within the local VA Medical Center and by direct mailings to veterans already included in a local research database. Research staff assessed study eligibility, conducted additional screening by telephone and electronic medical record review, and scheduled study visits with interested veterans. To recruit veterans with diverse perspectives, we stratified recruitment by whether the participant was a current firearm owner or former firearm owner or user. Of 119 male veterans who responded to study materials, 85 were able to be contacted and screened, 29 were eligible, and 17 consented and completed the study. Veterans who were screened and ineligible were screened out due to either exceeding the age criterion (n = 55) or being younger than the required age range (n = 1). In comparisons of eligible veterans who did (n = 17) and did not (n = 12) consent to participate in the study using measures available from the screening forms, there were no significant differences in age, firearm ownership, VHA eligibility, or self-reported mental health or memory problems. Veterans were compensated $40 for their participation. This study was approved by the Colorado Multiple Institutional Review Board.

### Procedures

**Fig 1:**
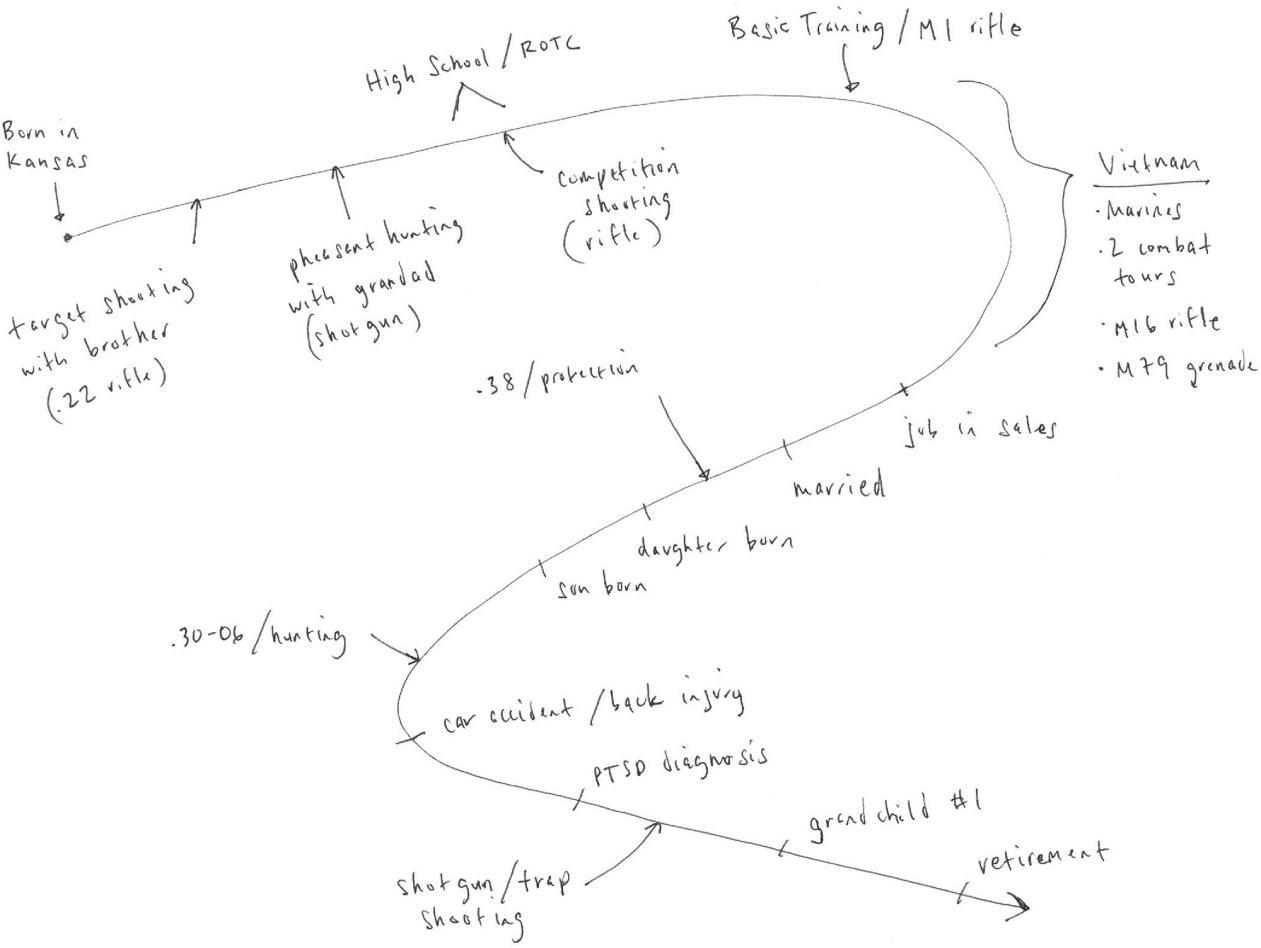
Fictional life timeline.

Study visits lasted approximately two hours and were conducted in-person by three interviewers (JAS, BDH, RH). Appointments began with informed consent, followed by semi-structured qualitative interviews, completion of self-report measures, and an interview regarding self-directed violence history (described below). The interview guide included open- and closed-ended questions developed by three investigators (JAS, BDH, LAB; included as an Appendix). Initial questions were designed to build rapport with participants by asking them to reflect on their military service. Subsequent questions were aimed at eliciting discussion relevant to firearm safety perceptions and lethal means safety interventions, including experiences using firearms, experiences or perceptions about firearm discussions with healthcare providers, and perceptions about the risks associated with firearm access. During interviews, participants completed life timelines in which they recorded and discussed major life events and how those events corresponded to their firearm-related experiences (see fictional exemplar created by JAS, Fig 1) [22]. Interviews were audio-recorded and transcribed.

### Measures

We assessed sociodemographic characteristics, military service history, and firearm-related characteristics via self-report, and recent mental health characteristics and trauma history using the Patient Health Questionnaire-9, PTSD Checklist for DSM-5, Alcohol Use Disorder Identification Test-Concise, Drug Use Disorders Identification Test, and VA Military Sexual Trauma screening questions [23–26]. Recent suicidal ideation and lifetime history of suicide attempt were also assessed, using the Adult Suicidal Ideation Questionnaire and Lifetime Suicide Attempt Self-Injury Interview, respectively [27, 28]. We reviewed the electronic medical record to assess whether each veteran recently (past 12 months) utilized VHA healthcare services.

### Data analysis

Prior to analysis, we engaged in bracketing to identify potential biases, their implications for data analysis, and strategies for managing potential biases during data analysis and interpretation [29]. We incorporated several methods to limit risk of bias in interpreting transcripts, including bracketing, triangulation, and the inclusion of a firearm owner and user on the study team. We performed thematic analysis using an inductive approach and conducted interviews until thematic saturation was reached [30, 31]. We (JAS, RH, LAB, LLM) reviewed transcripts and used analytic memos to note our initial impressions, significant quotations, and participants’ stories [32]. We used descriptive coding to assign labels to data by summarizing the basic topic of a passage using a word or short phrase. We met regularly to discuss salient themes that emerged from individual data and across groups. After several discussions about themes reflected in the data, we agreed that consensus had been reached. Rigor of data analysis was increased through researcher and theoretical triangulation [33]. Findings are reported according to The Standards for Reporting Qualitative Research (SRQR) [34].

## Results

The sample included one transgender and 16 cisgender male veterans with a mean age of 62.8 years (Table 1). Nine veterans identified as Black, six identified as White, and one identified as being of Hispanic ethnicity. Most were retired or unemployed. Over half reported a history of homelessness and all were recent users of VHA healthcare services. Participants had diverse educational and relationship histories, and had served in four armed service branches. Twelve veterans had been deployed and nine of those served on combat deployments. Additional sociodemographic and military service details are included in Table 1.

**Table 1:** Sociodemographic and military service characteristics.

	n = 17
Age, mean years (SD)	62.8 (5.2)
Gender, n (%)	
Cisgender male	16 (94.1)
Transgender male	1 (5.9)
Race, n (%)	
White	6 (35.3)
Black/African American	9 (52.9)
Multi-racial	2 (11.8)
Hispanic ethnicity, n (%)	1 (5.9)
Educational attainment, n (%)	
High school diploma or equivalent	1 (5.9)
Some college, no degree	7 (41.2)
Associate’s/bachelor’s degree	6 (35.2)
Master’s/doctoral degree	3 (17.6)
Relationship status, n (%)	
Married/cohabitating	10 (59.8)
Single	4 (23.5)
Widowed/divorced/separated	3 (17.7)
Sexual orientation, n (%)	
Heterosexual	16 (94.1)
Gay/queer	1 (5.9)
Children <18 years in household, n (%)	1 (5.9)
Employment status, n (%)	
Employed full- or part-time	3 (17.7)
Retired	6 (35.3)
Unemployed, not seeking employment	5 (29.4)
Unemployed, seeking employment	3 (17.7)
Lifetime history of homelessness, n (%)	10 (58.8)
Recent VHA healthcare use, n (%)	17 (100.0)
Military service branch, n (%)	
Army	5 (29.4)
Air Force	3 (17.7)
Navy	1 (5.9)
Marine Corps	4 (23.5)
Multiple^a^	4 (23.5)
Active duty service, mean years (SD)	5.7 (6.7)
Military service era, n (%) ^b^	
Vietnam	10 (58.8)
Post-Vietnam	10 (58.8)
Desert Storm / Desert Shield	3 (17.7)
OEF/OIF/OND	1 (5.9)
Highest rank, n (%)	
Enlisted	15 (88.2)
Non-Commissioned Officer / Officer	2 (11.8)
History of deployment, n (%)	12 (70.6)
History of combat deployment, n (%)	9 (52.9)

Abbreviations: *VHA*, Veterans Health Administration; *OEF*, Operation Enduring Freedom; *OIF*, Operation Iraqi Freedom; *OND*, Operation New Dawn

^a^ Veteran served in more than one military branch

^b^ Veterans may have served in more than one era

Three participants screened positive for probable depression, and three screened positive for probable posttraumatic stress disorder (Table 2). Two reported a history of military sexual trauma and two reported clinically-relevant suicidal ideation in the preceding month. None screened positive for unhealthy alcohol use or drug use disorder, and none reported a prior suicide attempt.

**Table 2:** Mental health characteristics.

	n = 17
	n (%)
Probable depression	3 (17.7)
Probable posttraumatic stress disorder	3 (17.7)
Positive screen for unhealthy alcohol use	0 (0.0)
Positive screen for drug use disorder	0 (0.0)
History of military sexual trauma	2 (11.8)
Clinically-relevant suicidal ideation	2 (11.8)
Lifetime suicide attempt(s)	0 (0.0)

Ten participants reported currently owning firearms (Table 3). Of the remaining seven, one was a former owner who currently resided with a firearm owner, five were former-owners who currently resided in households without firearms, and one was a former firearm user who had never owned a firearm and did not reside in a household with a firearm. In the prior 30 days, eight had handled a firearm, three had discharged a firearm, and two had carried a loaded handgun. Additional details regarding firearm storage practices are included in Table 3.

**Table 3:** Firearm characteristics.

	n = 17
	n (%)
Firearm ownership	
Firearm owner	10 (58.8)
Non-owner, resides in firearm household	1 (5.9)
Past owner, no household firearm	5 (29.4)
Never owner	1 (5.9)
Firearm stored in or around household^a^	9 (81.8)
Firearm stored locked	
All of them	5 (55.6)
Some of them	0 (0.0)
None of them	3 (33.3)
Unsure	1 (11.1)
Firearm stored loaded	
All of them	3 (33.3)
Some of them	1 (11.1)
None of them	4 (44.4)
Unsure	1 (11.1)
Any firearm stored loaded and unlocked	1 (11.1)
Ammunition stored locked	
All of it	5 (55.6)
Some of it	0 (0.0)
None of it	2 (22.2)
Unsure	0 (0.0)
No ammunition in household	2 (22.2)
Handled firearm in last 30 days	8 (47.1)
Discharged firearm in last 30 days	3 (17.7)
Carried loaded handgun in last 30 days	2 (11.8)

^a^ Denominator includes firearm owners and non-owners residing in households with firearms (n = 11)

### First firearm experiences

Nearly every veteran in our sample described having experiences with or using firearms at a young age. These experiences usually occurred with family members and, in all cases, with older males. One veteran reported receiving his firearm as a gift from his brother, stating, *“Oh*, *probably around 10 years old*, *because I grew up on a farm and we had rifles and shotguns*. *So it wasn’t a big deal*.*”* Another described discharging a firearm for the first time at the age of *“5 or 6*, *somewhere in that range*.*”* He was taught to handle the firearm *“probably (by) my uncle or dad*.*”* This veteran went on to describe the presence of firearms in his childhood home: *“Yeah*, *we always had a* .*22 (caliber) or a shotgun around*.*”*

Participants commonly described growing up in rural areas or on farms, where using firearms for hunting or target practice was routine. A veteran recalled:

*“Back in the ‘50s everybody went out in the woods and went hunting*…*a lot of kids in high school would have a gun rack in the back of their car*, *or just have the guns laying in their car because they’re going hunting either before or after school*. *Running out to my cousin’s house*, *I’m going to stop and do some hunting on the way*. *You know*, *it literally*, *it wasn’t a big deal*. *Everybody had them and everybody is aware of them*.*”*

### Early safety lessons

When asked about the most important firearm safety lesson learned during his initial experiences with firearms, one veteran responded, *“Probably safety…Just where you’re pointing it*.*”* Another veteran said, *“You had to always not point that weapon toward nobody* …*always keep it pointed up*, *point up*.*”* Another veteran was taught, *“Don’t fire anything if you don’t know what’s behind it…You always carry it barrel up*, *not loaded unless you’re out hunting*. *Then it’s always barrel down so if it discharges it’s in the ground*.*”* A veteran who grew up hunting with family offered a detailed explanation of the safety lessons he learned from his grandfather:

*“Before he handed me the bullet*, *he taught me gun safety*, *taught me how to hold the weapon*, *how to carry it*, *where to look at*, *he’d make sure I had my reflective vest on and everything else and we just waited*. *Be aware of your surroundings*. *Be aware of other hunters that may come to your line of sight*. *Make sure what you’re shooting at*, *you hit*. *And I was always terrified of missing because I’m thinking this round is going to hit somebody and kill them or maim them or whatnot*.*”*

While many participants described learning about how to handle firearms (e.g., where to aim), only one mentioned learning about the importance of where firearms should be stored when not in use:

*“And safety*, *of course*. *How to store it*, *keep it locked*, *and it’s just relative basic common-sense safety measures*…*In a gun case*, *a locked gun case* …*in the home in the closet*.*"*

However, many emphasized the importance of storing firearms unloaded when not in use. For example, one Veteran stated, *“Not to leave the gun loaded*. *Double check that it was empty*. *I would say that’s most important*.*”* Similarly, several reported the importance of assuming a firearm was loaded when handling it. Another veteran said, *“Make sure the gun is unloaded before you do anything with it…always considered (it) loaded*, *always consider your gun loaded*.*”*

When asked about early firearm safety lessons, veterans frequently mentioned the importance of “*common sense*” in preventing firearm injuries.

*“It’s just common-sense stuff*. *I mean even as a little kid I said*, *well duh*, *this is kind of stupid*. *Do we really have to*? *Do people actually shoot if they don’t know what’s behind it*?*”*

### Social value across the lifespan

Another theme among participants was the social value of firearm use with friends, family members, or coworkers across their lifespans. For example, despite not considering himself a firearm “*enthusiast*,” one veteran described target shooting as a way to bond with his father:

*"I wasn’t really an enthusiast for going out and shooting*. *I just would do it on a couple occasions just to*, *you know*, *do something with my dad*.*”*

Another veteran stated, *“A couple of coworkers had gone jack rabbit hunting out in (location)*. *And so they said come on…come with us*. *I guess we had talked about gun ownership and they knew that I had a few*, *I think then just* .*22 caliber*, *and for jackrabbits that’s fine*.*”*

### Protection

Veterans reported owning firearms for a variety of reasons across their lifespans, including personal and household protection, target or competitive shooting, hunting, as sentimental tokens, collector’s items, for employment (e.g., law enforcement, security), or as financial investments. However, regardless of their reasons for ownership, a common theme in this sample was the perceived utility of using firearms for protection. A firearm-owning veteran simply stated, “*A gun to me is for self-defense*.” Another veteran, who did not currently own a firearm, acknowledged that firearms could be useful in certain circumstances:

*“I can see some scenarios where*, *yeah*, *it’d be nice to have a gun if I was being robbed or if somebody broke into my house or*, *it’d be nice to have a gun to take care of the situation*. *I could see myself using a gun if somebody was trying to hurt my family—say rape my daughter or whatever*. *I think I could probably get pissed enough to say you don’t need to be alive anymore*.*”*

Another veteran described his reason for keeping firearms he inherited from his father:

*“My brother and I kind of split them up and at that time I didn’t have much interest in the rifles and just wanted a couple of them for self-defense at home*. *So*, *I thought that being able to protect myself was something that was*, *at that point in time*, *worth it*. *I mean my other weapons that I had for self-protection were knives so I just figured a couple guns wouldn’t hurt*.*”*

Another veteran also acknowledged that firearms could be useful *“for protection*,*”* but indicated that he did not need a firearm because he did not *“live in a bad neighborhood*.*”* Further illustrating veterans’ perceptions regarding the importance of having firearms for protection, one veteran attempted to teach his wife to use firearms in anticipation of his deployment:

*“(I) tried teaching my wife how to shoot*. *And I said hey*, *I’m going to be deployed*, *I’m worried about you*…*and she was always about no*, *I don’t want to touch the gun*, *I don’t want to shoot a gun*, *don’t come near me with a gun* …*I was just concerned with every day being able to*, *saying like hey*, *I’m the man in the house*. *But the man of the house is not going to be here*, *so you need to learn how to defend the homestead*.*”*

Veterans also stressed the importance of keeping a firearm readily accessible for protection. One said, *“For a gun to be any good to have*, *you to have it right on you where you could get at it quickly…if somebody’s after you*, *they’re not going to wait for you to go get your gun*.*”* Another veteran, who slept with a firearm in his bedside drawer, said, *“…in case anybody decides to try to get in my house*.*”* Similarly, a veteran who kept his firearm loaded described his reason for doing so: “*Why have it if it’s not loaded*? … *Safety off…Because I don’t want to sit there fumbling around if somebody comes through the window or comes through the door…*.*”*

A few veterans discussed their desire to own a firearm for protection in relation to aging and decreased physical health. One said, *“Yeah*, *because I’m older now…I lost a lot of the strength*. *I don’t have the strength or ability to protect myself right now*. *You know*, *as I did when I was younger*. *When I was younger*, *I didn’t really care*, *you know*? *Figured if I couldn’t fight it*, *I could run*. *Now I can’t do either*.*”* Another veteran stated, “*I know that 80% of the Vietnam Vets that I know here have weapons…for us it’s protection*. *We’re getting old and we don’t have the faculties that everybody has…depending on what neighborhood you go into*, *you’re easy prey*.”

Several veterans described why they felt firearms were necessary or useful for protection. Examples included prior experiences of being robbed, military service, criminal activities, for employment, and “*living in a bad neighborhood*.” For example, a veteran stated, “*When I was in (city)*, *I got robbed*. *That’s why I bought it*.” Another veteran indicated that he kept his firearm in his bedside drawer “*in case anybody decides to try to get in my house… I was a cop*, *you make a lot of enemies*. *I put a lot of people away in jail and they don’t all stay there*.” One veteran, who previously sold illicit drugs, said, “*When you’re dealing drugs and you involve that kind of money*, *you know*, *you got to carry something*.” Another said:

“*I’ve slept with a gun under my pillow before when I was younger*, *but that’s because I was in a bad neighborhood…I was living in [neighborhood] in a trailer court and it was a bad*, *bad neighborhood*…*a lot of gangs and stuff like that*. *It was just dangerous…*” He then said, “*But if you’re in a regular suburban house sitting there with a loaded gun*, *just sitting there just waiting*, *watching TV with a loaded gun right there*, *that’s what I call paranoid*. *Maybe I was a little paranoid too*. *Or scared*, *either one*. *But that was the neighborhood back then*.”

In some cases, veterans suggested that their fear of victimization was disproportionate to their actual risk. One veteran said:

*“I probably have some disproportionate issues with thinking that I’ll be robbed or have a home invasion*, *and I think it’s primarily gotten worse because I got robbed*…*I think my fear of it happening is greater than the actual chance of happening*. *So*, *I feel my fear is out of proportion for reality*. *I don’t live in a neighborhood where that’s going to happen realistically*. *I mean*, *yeah*, *it could happen*. *It could happen anywhere*. *But I’m not in the neighborhood where that tends to happen at all*. *And so I don’t see the*, *I don’t see where my fear is necessarily justified*. *It’s not like I’m living in a slum where people are shooting each other on the streets every other night*. *So*, *as I said*, *it seems to be disproportionate*.*”*

Similarly, another veteran owned a firearm for protection, but also acknowledged the relative safety of his neighborhood:

*“I’ve been living at this residence*…*it’s a family-oriented community and*, *I mean*, *nothing happens in our community*. *We all know each other’s kids and everything else like that*. *Everybody’s like really*, *you know*, *on the up and up*. *So there was*, *there’s never speak of no crime in that neighborhood*.*”*

### Risks of firearm access

Every participant reported at least one circumstance in which he felt that others should not have access to a firearm. Examples included those perceived to have an increased risk for suicide, depression, a history of violence, cognitive impairment, substance use, anger issues, readjustment challenges after military deployment, or those with insufficient proficiency in using firearms. For example, one veteran described a broad range of mental health factors that should be considered:

*“It’s your right supposedly to own a firearm*, *but everybody*, *everybody isn’t mentally capable*…*of owning and using a firearm*.*…People with mental issues*, *bipolar and stuff like that where your mood…can switch and change like*, *at a moment’s notice*, *should not have access to firearms*. *People get depressed…they have access to a firearm*, *they could hurt themselves…they have a tendency they might want to take themselves out*.*”*

Another veteran said, “*I think if I got severely depressed*, *which I don’t think I’m going to because I’m dealing with it*, *I’d probably think the gun should not be in the house*.” Another veteran felt that a history of violence should preclude firearm ownership: “*If you’re really a violent criminal*, *you shouldn’t own a gun*. *I mean if you got a history of violence*, *you shouldn’t own a gun*. *Whether it’s domestic violence or anything like that*. *Otherwise*, *as long as you don’t have no felonies*, *I see nothing wrong with it*. *Just as long as it’s not a military style weapon*.”

Regarding alcohol use, one veteran simply stated, *“Alcohol and bullets don’t really go together*.*”* Similarly, another veteran said, “*Because it’s drinking*, *and you can’t really be careful and drink*.*”* Another veteran reflected on his history of drug use: *“Because if you’re using chemicals like cocaine or meth you’re not in your right mind*. *I may have thought I was back then*, *but I wouldn’t have hesitated to shoot somebody back then either*.*”* One veteran, when discussing the challenges of reintegration after military deployment, said, *“I don’t think veterans who just came off deployment and just like me came out of the military should own a weapon*. *I think it’s too many pitfalls to fall into that we don’t realize*.*”*

Some veterans described scenarios in which they acted to limit a peer’s or family member’s access to firearms. One veteran firearm owner recalled regularly hiding his father’s firearms to prevent his father’s suicide or unintentional injury:

*“And my mother didn’t feel safe with the guns in the house and him drinking*. *So*, *since she knew I knew the combination (to the safe)*, *she would tell me go downstairs and get the weapons and hide them*. *You know*, *put them in the attic*, *something like that*. *And then lock it up*, *and then when he’s sober*, *we’ll put them back*.*”* One of this veteran’s final actions prior to joining the military was to give his father’s firearms to the police without his permission.

Another Vietnam-era veteran described how, as an adult, he intervened to limit firearm access by a fellow veteran in emotional crisis:

*“I have one friend*, *Vietnam Vet*, *he’s still around because I did take his gun away from him*.*”* When asked how, he said, *“Well it took about a couple hours*. *Just sitting there talking to him about things*.*”* He then, *“just took it away from him*.*”* When asked how that veteran responded to having his firearm taken from him, he said, *“He was pissed at first*.*”* But, *“…it’s a brotherhood*.*”* When asked if the veteran was still upset with him, he said, *“No*.*”*

### Lethal means safety discussions

When participants discussed who should be involved in conversations to limit their access to firearms if such access ever became unsafe, most referenced family members. One veteran stated, “*Probably the wife*, *but I think the number one would be me*.” Another veteran said, *“My daughter*. *I don’t have any issues with that*. *I trust her decision-making completely*.*”* One veteran mentioned various family members whom he would trust to initiate such a conversation, specifically referencing a time when they had acted to limit his access to his motorcycle for safety reasons, *“That’s what they told me about (my motorcycle)*. *I’d probably have to agree with them because…they’re family and they got my best interest in mind*.*”* A socially-isolated veteran said, “*It’s kind of a nice idea that somebody would be that much on your side to say let me take the gun*.*”*

Some veterans also indicated that healthcare providers can have a role in initiating discussions about firearm access. One veteran said, *“Well*, *if you give enough about me to say something or if you give enough about doing your job the right way*, *you would say something*. *You know*, *if you’re a professional in this field and you know somebody’s having issues and you don’t say something*, *you need your ass whooped*.*”*

However, many veterans felt that trust in the provider was an important prerequisite to discussing firearms with them. When asked how he might respond to a provider asking about his firearms, one veteran felt it would be appropriate because, ***“****Well*, *I think that my health team gives a pretty good crap about me*.*”* Conversely, lacking an established trusting relationship was considered a barrier to such discussions by another veteran, who stated, *“I don’t think people probably know enough about their physicians or the physician knows enough about them to be able to be involved in the decision*. *I think you have such limited exposure with your docs for the most part*.*”*

A few veterans described scenarios in which their providers had discussed firearm access with them:

*“It was okay*. *I mean I’ve been having this doctor for a long time*. *You know*. *So*, *me and her really got a good working relationship*, *where she’s one of the doctors that doesn’t hold her punches*. *She’s going*, *here it is*. *This is how it’s going to be whether you accept it or not*. *That’s the best medical advice for you*.*”*

Another veteran said that his mental health provider knew he had firearms at home. When asked how he would respond if she suggested changing his firearm safety behaviors, he responded, *“I have enough respect for her right now that I trust her judgement*. *I’ve known her now six years*, *seven years*.*”* However, he also reported reluctance to have the same conversation with his primary care providers, with whom he lacked established relationships:

“*It’s like here with the primary care physician*, *I’ve had five different ones this year*… *I’d tell them it’s none of their business*.*”*

## Discussion

The aim of this study was to understand older U.S. male veterans’ firearm-related beliefs and experiences to inform development and implementation of firearm-specific lethal means safety interventions.

This is one of only a few qualitative studies conducted specifically among veterans and members of the firearm-familiar community [17, 35]. Among male veterans, ages 50 to 70 years, who were current or former firearm owners or users, we identified several themes relevant for the development of firearm-related suicide prevention interventions.

Those interviewed used or learned about firearms early in life, usually through interactions with male family members. Firearms also served an important social function across participants’ lifespans. These findings highlight key differences between firearms and other lethal means (e.g., medications, sharp instruments). Early exposure to firearms, as well as the broader sociocultural context of their use, may serve as obstacles to behavior change, in that firearm owners may be reluctant to remove firearms from their households or to abstain from specific firearm-related activities. Removing a household firearm may partially isolate an individual from the social function of that firearm (e.g., sport shooting or hunting with friends). It is unclear whether that could contribute to social isolation, depression, or suicide risk, particularly for veterans who frequently engage in socialization that involves firearms. It may be important to improve our understanding of whether affecting firearm behaviors through lethal means safety interventions has unintentional costs, such as impacting belongingness or social connection, which are relevant to mitigating suicide risk [36]. Those developing lethal means safety interventions might consider strategies which effectively reduce household firearm access but allow for continued firearm-related socialization (e.g., storage of firearms at recreational facilities, such as shooting ranges). However, it’s also important to consider that these sites are common locations for firearm-related suicides.

Prior epidemiologic studies have shown that about two-thirds of firearm owners, including veterans, report personal or household protection as a primary reason for their ownership [3, 21]. In this sample, every veteran described firearms as useful for protection, regardless of their stated reason for owning firearms. In general, veterans in our sample described safety concerns as anticipatory (e.g., *“living in a bad neighborhood”*) or based on past experiences, rather than stemming from a current or recent experience (e.g., being assaulted). Their perceptions regarding the protective value of firearms (e.g., ownership and access) may have been influenced by prior stressful or traumatic experiences, which appeared to be relatively common in our sample (e.g., combat exposure, residing in dangerous neighborhoods). This is consistent with prior work showing that exposure to stressful or traumatic experiences can alter one’s sense of safety and lead to engaging in behaviors to increase sense of safety (e.g., obtaining firearms; storing firearm loaded and nearby) [37]. This finding reinforces another unique challenge to affecting changes in firearm-related behaviors. When recommending lethal means safety behaviors (e.g., storing firearms locked and unloaded), clinicians must be prepared to discuss balancing the benefits of such behaviors in reducing suicide risk against perceived increases in vulnerability to others. For example, behaviors such as storing firearms loaded and/or unlocked may increase a veteran’s sense of safety with respect to protecting him or herself and one’s family from others, but are also associated with increased suicide risk for those residing in the household [4, 5]. Incorporating specific communication strategies, such as motivational interviewing [38], into firearm lethal means safety interventions may be valuable in supporting patients in balancing these factors. Unfortunately, there are little objective data available to inform such conversations, as the prevalence of defensive firearm use and the effectiveness of those actions in preventing victimization remain under-researched [39]. Developing strategies to address these safety-related beliefs may be critical in facilitating effective firearm-related safety discussions. For example, cognitive reframing (i.e., identifying more accurate safety-based beliefs) or exposure-based strategies (e.g., assessing safety with a firearm stored unloaded or using a firearm lock) could be useful tools within the context of a therapeutic relationship (e.g., outpatient psychotherapy).

We enrolled veteran males ages 50 to 70 years given the high burden of suicide among this group [40]. This approach also allowed us to explore firearm-related experiences across their lifespans. Some participants reported that age-related declines in physical function were a key reason for feeling vulnerable and wanting a firearm. This is concerning, as age-related declines in mental (e.g., cognition) or physical functioning (e.g., stability, coordination) could place these individuals at greater risk for unintentional injuries or self-directed violence. Future work may be valuable in assessing the impact of aging on safety perceptions, and whether perceptions of vulnerability could be addressed by means other than firearms.

When asked about firearm safety lessons learned during early firearm encounters, nearly all participants described the importance of safe firearm handling and using “*common sense*” to prevent unintentional injuries. This is consistent with findings from a nationally representative study of U.S. adults conducted in 2015, which found that, of those who had received formal firearm training, most training topics focused on safe handling, storage practices, and prevention of unintentional injuries [41]. Among firearm owners, only 15% had received training in firearm-related suicide prevention. Similarly, when recounting important lessons learned about firearm safety, none of the veterans in our sample described learning about specific methods for preventing suicides by firearm. This finding is highly relevant to lethal means safety interventions. Perspectives regarding what it means to use a firearm “*safely*,” often learned and potentially solidified early in life, may be very different from what many clinicians might mean by “*firearm safety*” when discussed within the context of suicide prevention. These findings underscore the importance of identifying the most acceptable and effective language to be incorporated into lethal means safety interventions with veterans.

Every veteran we interviewed identified specific circumstances in which they perceived that individuals should not have access to firearms, and some described past scenarios in which they acted to limit someone’s firearm access. This is consistent with prior work showing that some veterans are willing to act to limit firearm access when a household member has elevated suicide risk [10]. Our findings also helped to clarify prior research by providing insight into factors important for initiating firearm-related conversations [15–18]. For male veterans in this age group, trust appears to be paramount [35]. This finding is consistent with prior studies demonstrating that acceptability, credibility, and trust are interrelated, foundational elements of firearm-related interventions, and suicide prevention interventions, generally [35, 42].

Some veterans in our sample described a willingness to openly discuss firearm safety–albeit specifically within the context of a trusted clinical relationship. While this is encouraging information for providers working with patients within such relationships, this presents a challenge to providers working with patients with acute suicide risk who may be charged with affecting firearm behaviors in the short-term, and in the absence of an established relationship (e.g., emergency department clinicians). Importantly, most veterans in our sample stated a clear preference for family to be involved in such discussions. It may be critical for future research to identify when and how to incorporate family members into firearm-related interventions, and how clinicians may meaningfully engage veterans outside the context of established relationships.

Several veterans in our sample discussed prior scenarios in which they acted to limit someone else’s firearm access during a crisis. This is promising with respect to the potential acceptability of firearm-related interventions within the context of suicide prevention, and is consistent with prior survey studies [10]. Support for involving peers in behavioral interventions has increased, including within VHA. Incorporating veterans and other peers into firearm-specific interventions may be one avenue to overcoming mistrust that may preclude firearm owners from divulging sensitive information to clinicians and healthcare systems [43].

Though most U.S. veterans identify as non-Hispanic and White, 64% of our sample identified as Black or multi-racial, and one participant identified as Hispanic. It’s unclear how our findings may have been influenced by racial or ethnic differences in firearm-related experiences and ownership, or differences in trust of healthcare systems or providers [44–46]. Though our interview guide did not specifically explore issues related to race or ethnicity, several participants referenced their race as influencing their firearm-related experiences and perceptions. Future work aiming to explore how the perspectives and experiences assessed in this study may differ by race or ethnicity is critical to developing individualized and culturally-relevant interventions.

There are limitations of this study to consider. The generalizability of findings is limited given the qualitative design and that we enrolled a small sample of male veterans ages 50 to 70 years, residing in the Mountain West of the U.S., who were eligible to receive VHA healthcare. We incorporated several methods to limit risk of bias in interpreting transcripts, including bracketing, triangulation, and the inclusion of a firearm owner and user on the study team. However, the identified themes could have been affected by bias. Similarly, participants’ reports of lifetime and firearm-specific experiences were potentially subject to recall and social desirability biases.

## Conclusions

Qualitative methodology is a valuable tool for improving our understanding of participants’ experiences and perceptions in order to develop personalized, effective healthcare interventions. We identified themes that could be used to inform the development of firearm-specific lethal means safety interventions with older male veterans. Findings underscore potential obstacles to affecting changes in firearm-related behaviors in this population, including the sociocultural value of firearms, early firearm exposure, and the perceived utility of firearms for protection. However, we also identified factors that could potentially facilitate such interventions, such as including trusted individuals (e.g., family, trusted healthcare providers) and addressing safety perceptions that may accompany age-related declines in physical functioning. Future work should aim to identify how best to personalize firearm-related lethal means safety interventions to these participant characteristics and preferences.

## Supporting information

S1 Data (DOCX)
