# Correction: Firearm-related experiences and perceptions among United States male veterans: A qualitative interview study

**DOI:** 10.1371/journal.pone.0231493

**Published:** 2020-04-02

**Authors:** Joseph A. Simonetti, Brooke Dorsey Holliman, Ryan Holliday, Lisa A. Brenner, Lindsey L. Monteith

The third author’s name is spelled incorrectly. The correct name is: Ryan Holliday. The correct citation is: Simonetti JA, Dorsey Holliman B, Holliday R, Brenner LA, Monteith LL (2020) Firearm-related experiences and perceptions among United States male veterans: A qualitative interview study. PLoS ONE 15(3): e0230135. https://doi.org/10.1371/journal.pone.0230135
