# Supplementary material for: Firearm-related experiences and perceptions among United States male veterans: A qualitative interview study
Source: PLoS One. 2020 Mar 10;15(3):e0230135. doi: 10.1371/journal.pone.0230135 (PMC7064196; doi:10.1371/journal.pone.0230135)
Supplement: S1 Data — (DOCX) [file pone.0230135.s001.docx]

**#17-0535: Semi-Structured Qualitative Interview**

**Interviewer Codes:**

*Italicized orange font Instructions to interviewer*

*Italicized black font Read (or paraphrase) to the participant*

**[blue, bold, and in brackets] Indicates information attempting to assess**

[black and in brackets] Modify wording to fit participants’ experiences.

When possible, use their language.

Similarly, the wording may have to be revised slightly based on what experiences the participant reports.

**START TIME: ______________________**


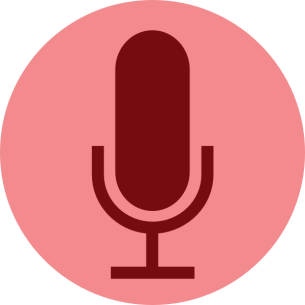
**REMEMBER TO RECORD!**

*In this interview, I will be asking you some questions about gun ownership and storage practices during your lifetime. For the first part of the interview, I will be asking you to tell me about your first experience handling a gun. After that, we will move on to talking about how guns fit into major aspects of your life. We will wrap up our interview by talking about gun safety and by talking about your thoughts on how VA providers should discuss gun access and safety with Veterans. Some of these questions may be upsetting, so please let me know if you would like a break.*

**MILITARY SERVICE**

1. Tell me about your experience of being in the military.

**[General experience of being in the military / ice breaker]**

**GUN HISTORY**

1. Can you tell me about the first time you shot a gun? **[First experience]**
   1. What kind of gun was it? **[Pistol, rifle, etc.]**
   2. When did this happen? **[Date, age, or year]**
   3. Where did this occur? **[Location]**
   4. Who taught you? **[Relationship to person]**
   5. What was the most important lesson that person taught you about *handling or owning* a gun? **[Firearm knowledge gained]**

**TIMELINE OF LIFE EVENTS**

1. Starting as far back as you can remember, I would like you to draw a timeline showing the major aspects of your life. This is an example of a timeline (show participant sample timeline). Some examples of things you could include in your timeline include:
2. Birth **[Date]**
3. Age at leaving home **[Age of independence]**
4. Education **[High school and/or college]**
5. Employment/Retirement **[Employment history]**
6. Relationships, marriage, birth of children **[Family events]**
7. Military service, deployment, injury, separation, combat **[Military related events]**
8. Location (city/state) **[Location or relocation]**
9. Health (e.g., depression, PTSD, physical illness) **[Physical and mental health]**
10. Now I would like you to go back to the beginning of your timeline and tell me how and where guns fit into your life.
11. First gun owned **[Type of gun and age acquired]**
12. Type of guns you’ve owned **[handgun, rifle, etc.]**
13. A Time that you have gotten a new gun or guns **[Purchased or given]**
14. When and why you got rid of guns **[Sold, lost, stolen]**
15. Why did you decide to get guns **[Motivations]**
16. *How do you or did you* use your guns **[Purpose]**
17. Storage practices of each gun with emphasis on reasons for change in practices **[Storage location and changes over time]**

**FIREARM SAFETY**

1. Can you tell me about a time you’ve been around someone handling a gun when you were concerned about that person’s (or your) safety. **[Safety issues]**

**□ Yes □ No** *(If No, proceed to Q6)*

1. Why were you concerned? **[Reason for concern]**
2. What did you think might happen? **[Worst fear]**
3. What did you do? **[Response to concern]**
4. Have you ever been around someone handling a gun who was having a difficult time in life? **[Safety issues] □ Yes □ No** *(If No, proceed to Q7)*
5. Can you describe what was making their life difficult **[Circumstances]**
6. *Can you describe a time or* *any other* scenario when you might be around someone handling a gun when you would be concerned about their safety or yours? **[Safety issues]**
7. Can you describe a time or scenario when maybe you shouldn’t have access to a gun? **[Safety issues] □ Yes □ No** *(If No, proceed to Q9)*
8. What situations? **[Circumstances of example provided]**
9. What about if one of these situations were to happen to you?
10. Severe depression or other emotional problems **[Mental health problems]**
11. If you were having thoughts about hurting yourself or killing yourself **[Suicidal ideation]**
12. Drinking too much alcohol **[Alcohol abuse]**
13. Abusing drugs **[Drug abuse]**
14. Alzheimer’s disease **[Cognitive impairment]**
15. Thinking about harming someone **[Homicidal ideation]**

**DISCUSSING FIREARM ACCESS AND SAFETY WITH VA**

1. *Under those circumstances or in the situation you described*, who would you want to bring this up with you? **[Relationship to person]**
2. How do you think you would react if your physician or health care provider brought this up with you? **[Response to discussion]**

**CONCLUDING REMARKS**

1. If there anything you would like to add to what we’ve discussed or anything you thought we would discuss today that we didn’t cover? **[Concluding remarks]**

**END TIME: ________________**


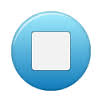
**STOP RECORDING**
